# Frontoparietal intraparenchymal hemorrhage secondary to anticoagulation

**DOI:** 10.1186/s12245-024-00723-0

**Published:** 2024-09-30

**Authors:** Savitra Ward, Benjamin Colaco Jamal, Latha Ganti

**Affiliations:** 1 Trinity Preparatory School, Winter Park, FL USA; 2 Brown University, Providence, RI USA; 3 Orlando College of Osteopathic Medicine, Winter Garden, FL 34787 USA; 4 Warren Alpert Medical School of Brown University, Providence, RI 02903 USA

**Keywords:** Intracererbal hemorrhage, Frontoparietal hemorrhage, Warfarin-associated bleed

## Abstract

The authors present the case of a patient experiencing frontoparietal intraparenchymal hemorrhage. With a history of a mechanical heart valve due to rheumatic disease, the patient was on warfarin and experienced a warfarin-associated bleed. The new 2002 guidelines for the management of intracerebral hemorrhage are discussed in the context of this case.

## Introduction

A hemorrhagic stroke is a neurological event characterized by bleeding within the brain tissue resulting from the leakage or rupture of a blood vessel [1]. There are two main types: intracerebral hemorrhage (ICH), which is bleeding into the brain tissue, and subarachnoid hemorrhage (SAH), which is bleeding into the space between the arachnoid and pia mater [2]. An intraparenchymal hemorrhage is a form of ICH that occurs within the brain parenchyma, the functional brain tissue that contains neurons and glial cells. Hemorrhagic strokes are the deadliest form of stroke, often connected to high mortality and morbidity [3]. The longer the stroke is allowed to progress, the worse the outcome. Because of this, early recognition, diagnosis, and treatment are essential. Out of the 15 million strokes per year, hemorrhagic strokes account for about 10–20% of them. In the United States, United Kingdom, and Australia, hemorrhagic strokes occur at a rate of 8–15%. This rate is higher in Japan and Korea at 18–24%. The global incidence ranges from 12 to 15% out of 1,000,000 [4]. The incidence rate for hemorrhagic strokes has increased in low-to-middle-income countries and Asia. The leading cause of hemorrhagic strokes is hypertension. Other significant risk factors include brain aneurysms, use of anticoagulants, family history, and increased age. Patients with hemorrhagic stroke often present with the following symptoms: acute onset headache, neck stiffness, vomiting, elevated blood pressure, and rapidly developing neurological signs [5]. When encountering left frontoparietal intraparenchymal hemorrhage, it is essential to understand the function of the frontoparietal region of the brain. The frontoparietal region is the part of the brain that detects sensory information (primary somatosensory cortex) and directs body movement (primary motor cortex) [6]. If there is a hemorrhage and this area is damaged, then problems with awareness and touch sensation are expected.

## Case presentation

A 61-year-old female came into the emergency department (ED) with complaints of occasional headaches, sudden weakness, and clumsiness in her right hand. Starting a few days prior, she had driven several hundred miles with her husband. While driving, she noticed difficulty texting her daughter with her dominant right hand. The patient denied any fever, chills, chest pain, nausea, vomiting, diarrhea, abdominal pain, current headache, vision changes, weakness of her left side or her right leg, speech problems, or seizure activity. With a history of hypertension and paroxysmal atrial fibrillation, the patient also had a mechanical heart valve for mitral valve replacement secondary to rheumatic fever. She took baby aspirin and daily warfarin for this. Her past medical history also included sick sinus syndrome, for which she underwent a permanent pacemaker placement a decade prior. Her family history was positive for ischemic stroke and diabetes.

In the ED, a stroke alert was activated. Her vital signs included a temperature of 36.4 ℃, a pulse of 75 beats per minute, blood pressure of 126/58 mmHg, a respiratory rate of 16 breaths per minute, and oxygen saturation of 96%. She was alert, awake, and oriented upon physical examination and in no acute distress. She was pleasant and conversational, with normal mental status as well. Her neurological exam was unremarkable, with normal cranial nerve and cerebellar testing. Her Glasgow coma score was 15, and her National Institute of Health stroke scale score was 2. There were no significant motor or sensory deficits except for her right biceps, triceps, and handgrip strength being weaker than baseline.

**Fig. 1:**
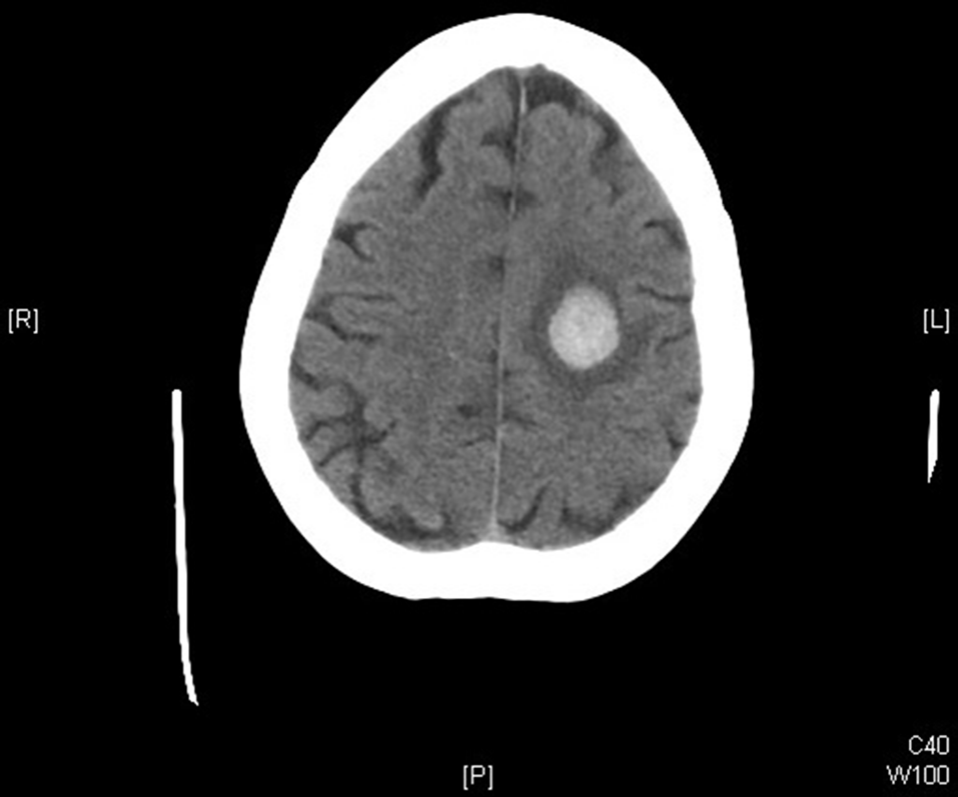
Non-contrast CT demonstrating left parietal lobe hemorrhage

Due to her past cardiac history, a cardiac evaluation was performed. The initial electrocardiogram (EKG) showed an electronic ventricular pacemaker at 75 beats per minute. Her cardiac enzymes were negative, along with the rest of her laboratory analysis. Next, a non-contrast computed tomography scan (CT) was obtained, revealing a rounded, well-circumscribed left parietal lobe hemorrhage measuring 2.3 × 1.7 × 2.7 cm with midline shift from left to right of 0.3 cm and moderate surrounding vasogenic edema (Fig. 1).

**Fig. 2:**
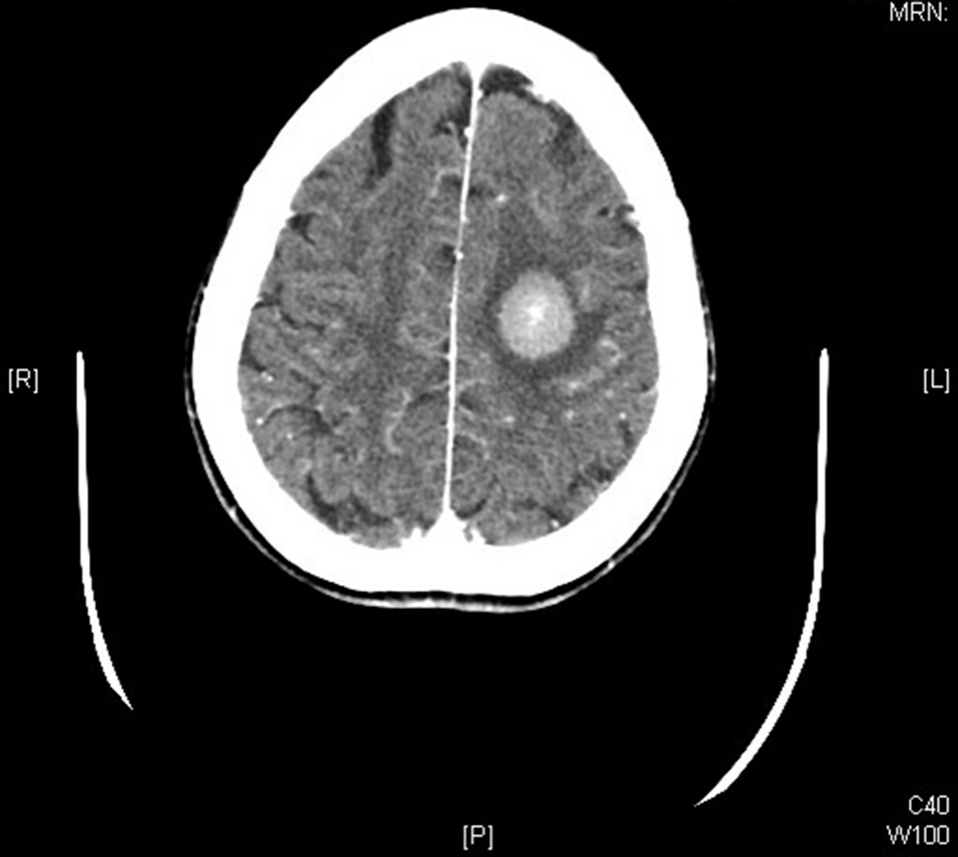
CT angiogram demonstrating intraparenchymal hemorrhage in the left parietal lobe

A CT angiogram confirmed the diagnosis of acute parenchymal hemorrhage in the left frontoparietal lobe (Fig. 2).

Calcified and soft plaque of the right carotid bulb and origin of the right internal carotid artery with approximately 70% stenosis were present. There was also calcified plaque at the left carotid bulb and origin of the left internal carotid artery with mild stenosis of approximately 25%. A contrast-enhanced MRI to exclude underlying neoplasm could not be done due to her mechanical heart valve being non-compatible with MRI.

The patient received one unit of prothrombin complex concentrate and intravenous vitamin K in the emergency department for the reversal of her warfarin-associated bleed. Neurosurgery was consulted, and no neurosurgical intervention was deemed necessary. She was admitted to the stroke service for risk stratification and secondary stroke prevention. Since the patient was stable, the plan was to observe her after the anticoagulation reversal for clinical or radiological signs of hematoma expansion.

The following day, a repeat brain CT demonstrated left frontal lobe white matter intraparenchymal hematoma but at a smaller size of 2.3 × 1.6 × 2.5 cm. She had become more stable from the previous day. An echocardiogram was performed, which revealed a normal ejection fraction and no acute abnormalities. Cardiology was consulted, and the pacemaker battery was still good for a year. They recommended resumption of her anticoagulation given her cardiac history and CHA₂DS₂-VASc score of 4 (1 point for hypertension, 1 point for female sex, and 2 points for history of stroke). The patient was discharged home on day 4 with instructions to resume her warfarin to keep her International Normalized Ratio (INR) between 2.5 and 3.0.

## Discussion

**Fig. 3:**
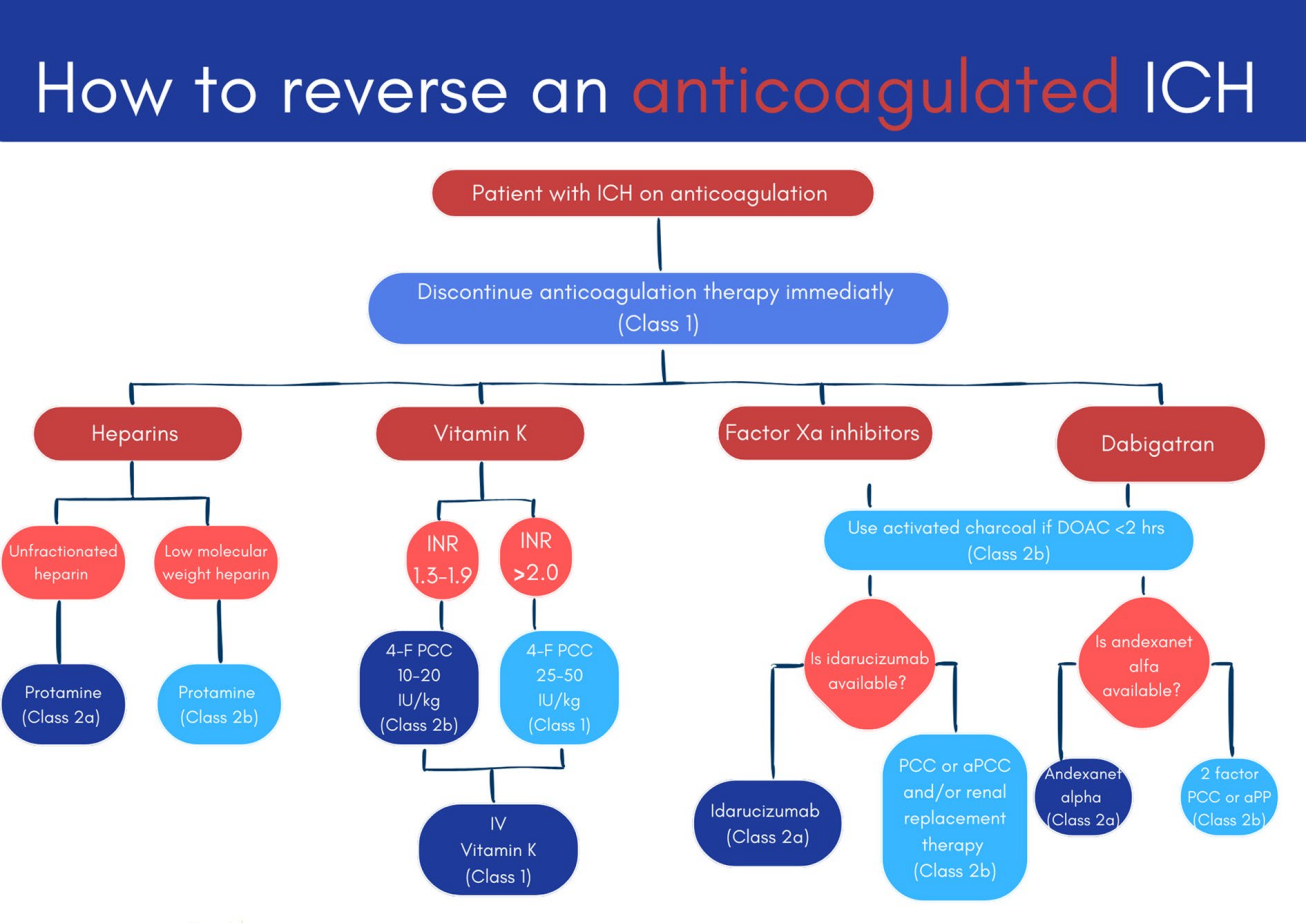
Infographic summarizing how to reverse various anticoagulated bleeds. Adapted from ASA guidelines [7], designed by Savitra Ward on Canva.com

In 2022, the American Stroke Association guideline for the management of Intracerebral Hemorrhage was updated to improve patient outcomes. A range of neuroimaging and clinical markers to predict the risk of hematoma expansion were recognized [7]. These guidelines emphasize the importance of rapid diagnosis, blood pressure control, and the reversal of anticoagulants [Fig. 3]. In this case, a patient who experienced a left frontoparietal intraparenchymal hemorrhage was treated following these new protocols.

The new guidelines begin diagnosis with signs detectable by a non-contrast computed tomography [7]. Noncontrast CT scans are the primary diagnostic tool used because of their speed and accuracy in detecting a hemorrhage [8]. In strokes, time is of the utmost importance. The quicker the diagnosis, the faster appropriate treatment can be administered, which gives the patient the best chance at a good outcome. In this case, upon arrival, the patient underwent a non-contrast CT scan, which confirmed the presence of a hemorrhagic stroke. The hemorrhage occurred in the frontoparietal region of the brain, which explains the patient’s symptoms. Her weakness and clumsiness in her right hand correlate to defects in the somatosensory cortex, which deals with touch [9]. Damage to the parietal lobe’s primary motor cortex, which deals with attention and awareness, can also explain the occasional headaches, as her brain must work harder to be aware and attentive.

Blood pressure management and anticoagulation reversal are other factors and essential components in dealing with ICH. High blood pressure can sometimes expand and worsen the bleeding [10]. Thus, the guidelines recommend maintaining a blood pressure of less than 140 mmHg by giving a blood pressure-lowering agent [11]. Additionally, because the patient was on warfarin, the guidelines recommended reversing her anticoagulation status to prevent further bleeding [7]. This was administered in the form of vitamin K, reversing the warfarin’s effects. This approach worked well. Through rapid diagnosis, blood pressure management, and anticoagulation reversal, this patient was stabilized and discharged in four days.

While reversing anticoagulation is well delineated, resumption of anticoagulation is more nuanced. This decision involves balancing the risk of subsequent ischemic stroke or other thrombotic event versus expansion or reoccurrence of the ICH. In our patient’s case, she remained stable without evidence of neurologic deterioration, and the hematoma size decreased. She also had a CHA₂DS₂-VASc score of 4, which is a strong indicator for continued anticoagulation, as it translates to a risk of 4.8% for ischemic stroke, and 6.7% risk of stroke/TIA/systemic embolism [12]. A 1 score for men or a score of 2 for women is “low-moderate” risk and anticoagulation should be considered; a score ≥ 2 for men or ≥ 3 for women is “moderate-high” risk and these patients should be on anticoagulation [13]. In the absence of expanding hematoma or other contraindications, these same cutoffs can be used for resumption of anticoagulation.

## Conclusion

This case of left frontoparietal intraparenchymal hemorrhage emphasizes the importance of following the new ICH guidelines. With rapid diagnosis and early intervention, these guidelines offer a standardized approach to managing these hemorrhages.

## Data Availability

No datasets were generated or analysed during the current study.
